# Individualized therapies in colorectal cancer: KRAS as a marker for response to EGFR-targeted therapy

**DOI:** 10.1186/1756-8722-2-18

**Published:** 2009-04-22

**Authors:** David Z Chang, Vikas Kumar, Ying Ma, Kuiyuan Li, Scott Kopetz

**Affiliations:** 1 Departments of Gastrointestinal Medical Oncology, The University of Texas, M.D. Anderson Cancer Center, Houston, Texas, USA; 2 Bellaire High School, Bellaire, Texas, USA

## Abstract

Individualized therapies that are tailored to a patient's genetic composition will be of tremendous value for treatment of cancer. Recently, Kirsten *ras *(KRAS) status has emerged as a predictor of response to epidermal growth factor receptor (EGFR) targeted therapies. In this article, we will discuss targeted therapies for colorectal cancers (CRC) based on EGFR signaling pathway and review published data about the potential usefulness of KRAS as a biological marker for response to these therapies. Results from relevant studies published since 2005 and unpublished results presented at national meetings were retrieved and summarized. These studies reflected response (or lack of response) to EGFR-targeted therapies in patients with metastatic CRC as a function of KRAS status. It has become clear that patients with colorectal cancer whose tumor has an activating mutation in KRAS do not respond to monoclonal antibody therapies targeting EGFR. It should now become a standard practice that any patients being considered for EGFR targeted therapies have their tumors tested for KRAS status and only those with wild-type KRAS being offered such therapies.

## Introduction

Over the past decade, we have witnessed an important development in the field of cancer treatment: therapy that is targeted to specific pathways involved in tumor growth and progression. This mechanistic, target-based approach is adding to the treatment options for cancer, and these treatments should be less toxic to normal cells and thus improve the therapeutic index.

To date, however, the overall effectiveness of targeted therapy in solid tumors has not been as robust as that achieved, for example, by Gleevec (imatinib) in the treatment of chronic myelogenous leukemia (CML). The difference in targeted therapy effectiveness in CML compared with solid tumors can be explained in part by the genetic etiology of the diseases. CML is caused by a single genetic alteration that results in a *BCR/ABL *fusion gene. This gene produces a chimeric protein with strong tyrosine kinase activity that can be effectively blocked by Gleevec. For most solid tumors, on the other hand, although they may appear to be morphologically similar on microscopic examination, molecular studies can identify different genetic alterations in tumors from different patients. Due to this heterogeneity, an agent targeting one particular pathway is unlikely to be effective in all patients. Clearly, there is a need to identify those patients who are most likely to respond to a specific therapy.

The identification of specific subgroups of patients who may benefit from a particular targeted therapy has been most successful in patients with breast cancer. Anti-estrogen treatment, an early type of targeted therapy, mainly benefits patients with estrogen receptor-positive breast cancer. Trastuzumab, a HER2-targeting monoclonal antibody, is most beneficial in patients with tumors that overexpress HER2. Recent data also suggest that genetic profiling can predict which patients may benefit from adjuvant therapy after resection of their breast cancers (e.g., Genomic Health's Oncotype DX^® ^test, which profiles the expression of 21 genes and makes a prediction about the likelihood of disease recurrence). These findings show great promise for identifying patients eligible for treatment with specific targeted therapies, as well as for making decisions about dosage and length of treatment.

Individualized therapies that are tailored to a patient's genetic composition and tests that can predict which therapy he/she will respond to will be of tremendous value for colorectal carcinoma (CRC). Despite significant progress in the development of new therapies over the last decade, CRC remains one of the top three causes of cancer death in the United States, where it is estimated that 148,810 patients will be newly diagnosed with CRC in 2008, with 49,960 deaths from this disease [1]. Many of these patients will receive one or more lines of chemotherapy, but not everyone responds to each regimen. For example, the targeted agent cetuximab as a single agent has a response rate of only about 10% in patients with irinotecan-refractory CRC [2,3]. In other words, the majority of people receiving cetuximab may not benefit from it, while incurring all the associated cost and toxicities. Considering the large number of cases of CRC, this translates into millions of dollars spent and significant toxicities experienced with no benefit.

In this article, we will discuss targeted therapies for CRC based on the epidermal growth factor receptor (EGFR) signaling pathway and review published data about the potential usefulness of the downstream oncogene Kirsten *ras *(KRAS) as a biological marker for response to these therapies. Results from relevant studies published since 2005 and unpublished results presented at national meetings were retrieved and summarized. These studies reflected response to EGFR-targeted therapies in patients with metastatic CRC as a function of KRAS status, and were divided into three groups: (1) previously treated patients who received cetuximab therapy; (2) previously treated patients who received panitumumab therapy; and (3) chemotherapy-naïve patients who received cetuximab therapy. Data retrieved included KRAS status (wild type [WT] or mutant type [MT]) and outcome (objective response rate [RR; complete response + partial response], time to progression [TTP], and overall survival [OS]). Descriptive statistics were used to compare outcomes in the three treatment groups as a function of KRAS status.

### Rationale of KRAS Status as a Predictor of Response to EGFR Targeted Therapy

#### The EGFR signaling pathway and targeted therapies for CRC

EGFR is a member of the HER (ErbB) family of human epidermal growth factor receptors that can promote tumor cell proliferation in a variety of epithelial malignancies. The EGFR molecules are 170 kd transmembrane glycoproteins: the extracellular domain has the ligand binding site and also contains specific sequences that are involved in dimerization, while the intracellular domain is a catalytic site that has tyrosine kinase activity. EGFR binds soluble ligands, including epidermal growth factors (EGFs) and transforming growth factor-alpha, and the ligand/receptor complex then signals the formation of receptor dimers (either homodimers or heterodimers with other members of the HER family). Dimerization triggers an intracellular phosphorylation cascade that transmits the original ligand-generated signal from the cell surface to the nucleus, causing downstream changes in gene expression that affect cell proliferation, migration, differentiation, and apoptosis. Overexpression of EGFR has been detected in many human cancers, including CRC.

Monoclonal antibodies that target EGFR can be effective as anticancer therapy in several ways. They can block ligand binding to the receptor, aborting the process of dimerization and phosphorylation that allows downstream signal transduction. In some cases, the antibody/receptor complex may also be immunogenic, leading to antibody-dependent cellular cytotoxicity (ADCC).

Two monoclonal antibodies that target EGFR have clinical activity against CRC: cetuximab and panitumumab. Cetuximab is a recombinant, chimeric, IgG1 monoclonal antibody, while panitumumab is a fully humanized IgG2 antibody. Because of their differing isotypes, it is possible that these two antibodies may differ in their mechanism of action, but this has not been documented. Cetuximab has been demonstrated to improve response rate, time to progression, and overall survival when added to irinotecan for patients with irinotecan-refractory metastatic CRC [2]. Both cetuximab and panitumumab have been shown to improve outcomes in patients with chemo-refractory metastatic CRC compared with best supportive care [4,5].

#### KRAS is an important molecule in the EGFR signaling pathway

KRAS encodes a membrane-associated GTPase that is an early player in many signal transduction pathways. KRAS acts as a molecular on/off switch for the recruitment and activation of proteins necessary for the propagation of growth factor and other receptor signals, such as c-Raf and PI 3-kinase. When activated, KRAS is involved in the dephosphorylation of GTP to GDP, after which it is turned off. The rate of GTP to GDP conversion can be sped up dramatically by an accessory protein of the Guanine nucleotide activating protein (GAP) class, for example RasGAP. KRAS can also facilitate the release of bound nucleotide by binding to proteins of the Guanine Nucleotide Exchange Factor (GEF) class, for example Soverall survival-1. HRAS is subsequently released from the GEF and quickly re-binds to the now available GTP resulting in HRAS activation.

When EGF ligand binds to the extracellular part of the EGFR receptor, the receptor dimerizes and its enzymatic activity is activated, resulting in phosphorylation of the intracellular domain. Subsequently, cellular effectors bind to phosphorylated residues of the intracellular domain and are activated, mainly through relocalization to the plasma membrane. When an activating mutation occurs in the KRAS gene, the RAS G-protein activates the mitogen-activated protein kinase (MAPK) signaling cascade downstream of EGFR. This may bypass the need for ligand binding to EGFR, conferring resistance to therapies like cetuximab or panitumumab which target the EGFR extracellularly.

Activating mutations of the KRAS gene have been widely studied as markers for cancer prognosis. These gene mutations, principally in codons 12 and 13, occur in up to one-half of CRCs, and population-based studies have suggested that the mutations might be associated with some tumor phenotypes [6].

### KRAS mutation predicts unresponsiveness to EGFR-targeted monoclonal antibody therapy in previously treated patients with metastatic CRC

#### The Data for Cetuximab

Table 1 summarizes the results of 12 studies in which previously treated patients with metastatic CRC received treatment with cetuximab alone or cetuximab as part of a multi-drug regimen (in combination with either irinotecan or oxaliplatin) [7-18].

**Table 1:** KRAS and treatment response to Cetuximab or Panitumumab in previously treatment patients with colorectal cancer.

**Study**	**Treatments**	**KRAS**	**N (%)**	**RR, N (%)**	**TTP (months)**	**OS (months)**	**Remarks**
***Cetuximab Studies***							
Moroni, 2005	CTX = 12	WT	16	6 (38%)	NA	NA	KRAS MT has worse RR.
	CTX + IRI = 9	MT	5 (24%)	0 (0%)	NA	NA	
Lievre, 2006^a^	CTX = 1	WT	17	11 (36.7%)	NA	16.3	KRAS MT has worse RR and OS.
	CTX + IRI = 25	MT	13 (43.3%)	0	NA	6.9	
	CTX + FOLFIRI = 4						
Benvenuti, 2007	CTX = 12	WT	32	10 (31.3%)	NA^b^	NA	KRAS MT has worse RR and TTP.^b^
	CTX + IRI = 11	MT	16 (33.3%)	1 (6.3%)	NA^b^	NA	
Finocchiaro, 2007	CTX = 5	WT	49	13 (26.5%)	6.1	10.8	KRAS MT has worse RR, TTP, and OS.
	CTX + IRI = 77	MT	32 (39.5%)	2 (6.3%)	3.7	8.3	
	CTX + OX = 3						
Di Fiore, 2007	CTX + IRI/OX	WT	37	12 (32.4%)	5.5	NA	KRAS MT has worse RR and TTP.
		MT	22 (37.3%)	0	3.o	NA	
Khambata-Ford, 2007	CTX	WT	50	6 (12%)	2.0	NA	KRAS MT has worse RR.
		MT	30 (37.5%)	0	2.0	NA	
Lievre, 2008^a^	CTX = 2	WT	65	26 (40.0%)	7.9	14.3	KRAS MT has worse RR, TTP, and OS.
	CTX + IRI = 78	MT	24 (27.0%)	0	2.5	10.1	
	CTX + FOLFIRI = 9						
DeRoock, 2008	CTX = 30	WT	67	27 (40.9%)	6.0	10.8	KRAS MT has worse RR, TTP, and OS.
	CTX + IRI = 83	MT	46 (40.7%)	0	3.0	6.8	
Tejpar, et al. 2008	CTX + IRI	WT	62	NA	NA	11.5	KRAS WT has worse OS.
		MT	33 (35%)	NA	NA	4.2	
Stoehlmacher, 2008	CTX + IRI/OX	WT	22	NA	NA	NA	KRAS WT did not respond.
		MT	8 (26%)	0	NA	NA	
Tejpar, et al. 2008^a^	IRI + CTX standard dose	WT	23	7 (30.4%)	NA	NA	KRAS MT has worse RR.
		MT	20 (44.4%)	0	NA	NA	
	IRI + CTX escalated dose	WT	31	13 (41.9%)	NA	NA	Escalated dose did not overcome KRAS MT.
		MT	12 (27.3%)	0	NA	NA	
Karapetis, et al. 2008	CTX	WT	117	15 (12.8%)	3.7	9.5	CTX also improved quality of life in KRAS WT patients.
		MT	81 (40.9%)	1 (1.2%)	1.8	4.5	
	BSC	WT	113	NA	1.9	4.8	
		MT	83 (42.3%)	NA	1.8	4.6	
***Panitumumab Studies***							
Moroni, 2005	PAN	WT	5	2 (40%)	NA	NA	KRAS MT did not have impact on response.
		MT	5 (50%)	2 (40%)	NA	NA	
Benvenuti, 2007	PAN	WT	15	3 (20%)	NA^b^	NA	KRAS MT has worse RR and TTP.^b^
		MT	10	1 (10%)	NA^b^	NA	
Amado, 2008	PAN	WT	124	21 (16.9%)	3.1	8.1	KRAS MT has worse RR, TTP, and OS.
		MT	84 (40.4%)	0	1.9	4.9	
	BSC	WT	119	0	1.8	7.6	
		MT	100 (45.7%)	0	1.8	4.4	
Freeman, 2008	PAN	WT	38	4 (10.5%)	4.1	10.7	KRAS MT has worse RR, TTP, and OS.
		MT	24 (38.7%)	0	1.9	5.6	

^a ^The 2006 and 2008 studies by Lievre and coworkers were based on independent patient series.

^b ^For all patients (CTX and PAN), average TTP was 3.7 months for patients with wild type KRAS versus 1.7 months for patients with mutant KRAS.

Abbreviations: CTX = cetuximab; PAN = panitumumab; IRI = irinotecan; Ox = oxaliplatin; Cap = capcitabine; BSC = best supportive care; WT = wild type; MT = mutant-type; RR = objective response rate (complete response + partial response); TTP = time to progression, OS = overall survival; NA = Not Available or Not Applicable.

Across all studies, approximately one-third of patients (median 36%, range 24% – 44%) had KRAS MT tumors. In the 10 studies that reported objective tumor response to cetuximab-containing therapy, the median RR in patients with KRAS WT tumors was 35% (range 12% – 42%) compared with 0% (range 0% – 6%) in patients with KRAS MT tumors. TTP was reported in 6 studies, with a median TTP of 6.1 months (range 1.8 months – 7.9 months) in patients with KRAS WT tumors compared with 3.0 months (range 1.8 months – 3.7 months) in patients with KRAS MT tumors. OS was reported in 6 studies, with median values of 11.5 months (range 9.5 months – 16.3 months) compared with 6.9 months (range 4.2 months – 10.1 months) associated with KRAS WT and KRAS MT, respectively.

In summary, all studies discussed above using cetuximab either as a monotherapy or in combination with either irinotecan- or oxaliplatin-based chemotherapy for previously treated metastatic CRC patients showed that KRAS mutational status clearly predicts unresponsiveness to cetuximab.

The study reported by Di Fiore and colleagues [11] underscores the importance of using sensitive molecular methods to ensure efficient mutation detection. In this study, 59 patients with previously treated metastatic CRC were treated with cetuximab plus either irinotecan- or oxaliplatin-based chemotherapies. Using direct sequencing of DNA extracted from tumor samples, the investigators detected a KRAS mutation in 16 out of 59 (27%) patients. Of these 16 patients, 13 had a progression of disease and three had stable disease. No KRAS mutation was found in the 12 patients with a complete or partial tumor response. The investigators then screened the tumors without detectable KRAS mutations, using two sensitive methods able to specifically detect KRAS exon 2 mutations: a multiplex SNaPshot assay based on primer extension able to detect the different KRAS mutations simultaneously in a single tube, and a fluorescent PCR-LCR assay. These two analyses were performed on samples from 11 out of 12 patients with either complete response or partial response, in 15 out of 16 patients with stable disease, and in 15 patients with disease progression, all of whom had no mutations revealed by direct sequencing of tumor DNA. Five additional KRAS mutations were detected by both methods and one mutation was detected only by PCR-LCR assay. These six additional mutations were found in two patients with stable disease and four patients with disease progression. SNaPshot and PCR-LCR assays confirmed the absence of KRAS mutations in the CR/PR patients. Therefore, in this series of 59 patients with metastatic CRC, sequencing analysis supplemented by SNaPshot multiplex and PCR-LCR assays led to the detection of a KRAS mutation in 22 samples (37%), rather than the 11 samples (27%) with direct sequencing alone.

Several studies have suggested that the predictive value of KRAS in determining response to cetuximab-containing regimens may be improved by combining it with other predictive factors. [15] determined the KRAS mutation status and mRNA expression levels of the EGFR ligands amphiregulin and epiregulin in 95 patients with primary CRC treated with cetuximab and irinotecan and correlated these variables with response and overall survival. They found that amphiregulin and epiregulin expression influenced RR and OS in patients with KRAS WT tumors, but not in patients with KRAS MT tumors, and concluded that the combined use of these markers may allow improved prediction of outcome to cetuximab plus irinotecan.

Khamabata-Ford and colleagues [12] attempted to systemically identify markers that are associated with disease control in patients treated with cetuximab as a monotherapy. The trial enrolled 110 patients with metastatic CRC who had received at least one prior therapy. Transcriptional profiling was conducted on RNA from mandatory pretreatment metastatic biopsies to identify genes whose expression correlated with best clinical responses. Consistent with the findings of Tejpar et al., they found that patients with KRAS WT tumors and patients with tumors that express high levels of epiregulin and amphiregulin are more likely to have disease control and increased TTP with cetuximab.

Stoehlmacher and colleagues [16] evaluated the predictive value of KRAS mutations and polymorphisms of EGFR and IgG-Fc-receptor in 40 patients with metastatic CRC receiving cetuximab-containing chemotherapy (in combination with irinotecan, FOLFIRI, or FOLFOX). They found that both KRAS and the EGF-A16G polymorphism significantly predicted response to cetuximab-containing treatment combinations, regardless of the specific regimen selected.

#### The Data for Panitumumab

The outcomes in 4 studies in which previously treated patients with metastatic CRC received treatment with panitumumab as monotherapy are similar to those reported above for cetuximab (Table 1). In the two largest studies [19,20], no patients with KRAS MT tumors showed an objective tumor response to panitumumab. In each case, KRAS MT was also associated with reduced TTP and OS. The data reported by Benvenuti and colleagues [9] showed a two-fold increase in RR associated with KRAS WT status compared with KRAS MT status (20% vs. 10%, respectively). KRAS MT status did not show an impact on tumor response in the report from Moroni and colleagues [7], but the patient numbers in this study were very low, with only 5 patients each showing KRAS WT and KRAS MT status.

In the report from Amado and colleagues [19], patient outcomes with panitumumab treatment were compared with outcomes in a matched population of patients who received best supportive care only. It is interesting to note here that KRAS status did not have an effect on TTP in the absence of treatment with the EGFR-targeting therapy, although KRAS MT appeared to be associated with reduced OS in both treatment groups.

Most recently, interim results from the PRECEPT study were reported at the 2008 ASCO annual meeting (data from final analysis not available for inclusion in Table 1) [21]. This phase II, open-label, single-arm trial was designed to prospectively estimate the efficacy of panitumumab plus FOLFIRI treatment as a function of tumor KRAS status in patients undergoing second-line treatment for metastatic CRC. A total of 110 patients with metastatic CRC with progression after first-line oxaliplatin-based chemotherapy plus bevacizumab were enrolled in this study. Patients received panitumumab and FOLFIRI every 2 weeks until disease progression or intolerability. Efficacy endpoints included objective response rate, progression-free survival, and overall survival by KRAS status. Data reported from the interim analysis supported previous studies showing that patients with KRAS MT tumors do not respond to panitumumab therapy.

In summary, as with cetuximab, all available studies (with the exception of the very small data set reported by Moroni et al. [7] with panitumumab as monotherapy or in combination with other agents for previously treated metastatic CRC patients show that KRAS mutation status clearly predicts response to panitumumab.

### KRAS mutation predicts unresponsiveness to EGFR-targeted monoclonal antibody therapy in first line treatments for metastatic CRC

The aforementioned studies demonstrated the predictive value of KRAS for outcomes of EGFR-targeted monoclonal antibody therapy in patients with metastatic CRC who had received previous chemotherapy. Does the predictive value of KRAS also apply to chemotherapy-naïve patients? At the 2008 ASCO annual meeting, at least four studies confirmed that it does. (Table 2) [22-25] Three of these studies, presenting results from the phase III CRYSTAL and CAIRO2 trials and the phase II OPUS trial, compared outcomes in patients treated with standard chemotherapy regimens (FOLFIRI, CapOxBev, FOLFOX) with or without the addition of cetuximab [22,24,25]. The fourth study compared an every-2-week schedule of cetuximab with the approved weekly regimen [23].

**Table 2:** KRAS and treatment response to Cetuximab-containing regiments in chemotherapy-naïve patients with colorectal carcinoma.

**Study**	**Treatments**	**KRAS**	**N (%)**	**RR, N (%)**	**TTP** **(months)**	**OS** **(months)**	**Remarks**
Bokemeyer, 2008	FOLFOX + CTX	WT	61	37 (60.7%)	7.7	NA	KRAS MT has worse RR and TTP. Cetuximab may have detrimental effects in KRAS MT.
		MT	52 (46.0%)	17 (32.7%)	5.5	NA	
	FOLFOX	WT	73	27 (37.0%)	7.2	NA	
		MT	47 (39.2%)	23 (48.9%)	8.6	NA	
Cervantes, 2008	CTX	WT	29	8 (27.6%)	NA	NA	Patients were treated with CTX first, then in combination with chemo. KRAS MT has worse RR and TTP.
		MT	19 (39.6%)	0	NA	NA	
	CTX + FOLFIRI	WT	29	16 (55.2%)	9.4	NA	
		MT	19 (39.6%)	6 (31.6%)	5.6	NA	
Punt, 2008	CapOxBev+CTX	WT	153	NA	10.5	22.2	KRAS MT has worse TTP, and OS. Cetuximab may have detrimental effects in KRAS MT.
		MT	93 (37.8%)	NA	8.6	19.1	
	CapOxBev	WT	152	NA	10.7	23.0	
		MT	103 (40.4%)	NA	12.5	24.9	
Van Custem, 2008	FOLFIRI + CTX	WT	172	102 (59.3%)	9.9	NA	KRAS MT has worse RR and TTP. Cetuximab may have detrimental effects in KRAS MT.
		MT	105 (37.9%)	38 (36.2%)	7.6	NA	
	FOLFIRI	WT	176	76 (43.2%)	8.7	NA	
		MT	87 (33.1%)	35 (40.2%)	8.1	NA	

Abbreviations: CTX = cetuximab; PAN = panitumumab; IRI = irinotecan; Ox = oxaliplatin; Cap = capcitabine; Bev = bevacizumab; BSC = best supportive care; WT = wild type; MT = mutant type; NA = Not Available or Not Applicable; RR = objective response rate (complete response + partial response); TTP = time to progression; OS = overall survival.

Across all of the treatment arms shown in the 4 studies in Table 2, a median of 40% of patients (range 33% – 46%) had KRAS MT tumors. For patients with KRAS WT tumors compared with KRAS MT tumors, the median RRs to cetuximab-containing treatment regimens (4 study arms reporting) were 58% (range 28% – 61%) and 33% (range 0% – 36%), respectively, and the median TTP values (4 study arms reporting) were 9.7 months (range 7.7 months – 10.5 months) and 6.6 months (range 5.5 months – 8.6 months), respectively. OS was reported in only one study [24], which noted a small increase in OS associated with KRAS WT compared with KRAS MT (22.2 months versus 19.1 months, respectively).

In summary, under controlled conditions in these studies of first-line treatment in metastatic CRC, KRAS mutation was found to be a predictive marker for lack of response to cetuximab treatment, either alone or in combination with irinotecan- or oxaliplatin-based chemotherapies.

It should be noted that, at least in the first line setting, there is a concern that cetuximab may actually have detrimental effects for patients with KRAS MT tumors. In the three studies designed to compare outcomes from standard treatment with or without cetuximab [22,24,25], patients with KRAS MT tumors showed a decrease in TTP in the cetuximab-containing arm compared with the standard treatment arm, although the difference in each individual study did not reach statistical significance. In the CARO2 study which compared CapOxBev with and without cetuximab, patients whose tumor has MT type KRAS did worse which did reach statistical significance [24]. Thus, not only does cetuximab appear to have no benefit in patients with KRAS MT tumors, it may have a negative effect on outcome, particularly in combination with bevacizumab and chemotherapy in first line treatment of metastatic colorectal cancer.

### KRAS and skin rash are independent predictive markers for response to EGFR-targeted monoclonal antibody therapies

Long before KRAS emerged as a predictive marker for responsiveness to EGFR monoclonal antibody targeted therapy, it was well known that skin rash was a very good surrogate marker for responsiveness to EGFR targeted therapies. An obvious question is whether skin rash and KRAS are independent predictors. The recent analysis of the KRAS data from the EVEREST study shed some light on this question [17]. In this study, patients with grade 0/1 skin reactions after 22 days of treatment with irinotecan and standard-dose cetuximab were randomized to receive a standard dose (250 mg/m^2^) (Arm A) or escalated doses (up to 500 mg/m^2^) (Arm B) of cetuximab. The results demonstrated that, in patients with metastatic CRC after failure of irinotecan-based therapy, treatment efficacy could be improved by escalating the dose of cetuximab in combination with standard-regimen irinotecan compared with standard-dose cetuximab for patients with grade 0/1 skin reactions. To determine whether dose escalation was also able to induce response in patients with mutated KRAS, the authors analyzed KRAS mutation status using archived tissue from 77 of 89 randomized patients. For patients on Arm A, who received the standard dose of cetuximab, response rates were 21.1% and 0% for wild-type KRAS and mutant KRAS, respectively. For patients on Arm B, who received escalated doses of cetuximab, the response rates for patients with wild-type KRAS and mutant KRAS were 46.4% and 0%, respectively. Therefore, improved response was mainly seen in the patients with wild-type KRAS tumors, while increased dose of cetuximab did not overcome reduced response in mutant KRAS tumors.

Although patients originally selected for randomization had a 0/1 skin reaction after 22 days of standard treatment, with continued treatment some patients developed a more severe cutaneous response to the medication. Skin reaction predicted clinical outcomes in patients with both KRAS WT and KRAS MT tumors, with a grade 2/3 reaction associated with improved progression-free survival (PFS) compared with a grade 1/2 reaction in each group. However, the overall range of PFS values was significantly higher in the KRAS WT group; at 200 days, the average PFS associated with a Grade 2/3 reaction was 60% in the wild-type KRAS group compared with approximately 34% in the mutant KRAS group. The ability of skin reaction to distinguish subgroups of KRAS WT or MT patients with differing outcomes argues that skin reaction and KRAS status are independent predictors of response to cetuximab-based treatment.

## Summary

Recent developments in individualized therapies for CRC are having a significant impact on current clinical practice and on the future development of treatments for this disease. To date, clinical activity has been assessed in over 2500 patients treated with cetuximab/panitumumab, either as a single agent or in combination with both FOFIRI and FOLOX chemotherapy, in both chemotherapy-refractory and chemotherapy-naïve settings. There is strong evidence that mutated KRAS in tumors predicts unresponsiveness to EGFR-targeted antibody therapies. Furthermore, data from the CRYSTAL, OPUS, and CAIRO2 studies showed evidence that the addition of cetuximab to chemotherapy (both oxaliplatin- and irinotecan-based) may have detrimental effects on patients with KRAS MT tumors. It is therefore important to test KRAS status in tumors of all patients being considered for EGFR-targeted antibody therapies, and only those patients with KRAS WT tumors should receive such treatments. Randomized prospective trials are not needed nor are they ethical to prove this concept further. In fact, the European Medicines Agency (EMEA) approved panitumumab only for patients with KRAS wild-type tumors. It is likely that verification of KRAS wild-type status will be required by the United States Food and Drug Administrative (US FDA) for cetuximab and panitumumab treatment in the near future.

Several commercial DNA sequencing-based KRAS tests for tumor tissues are available. Developing and validating more sensitive, reproducible, and affordable KRAS tests that can be used in the clinic is an important task that industry and government should undertake in order to maximize the value of this biomarker for individualized therapies in CRC.

## Future directions

Moving forward, there are several issues, both immediate and long term, that need to be addressed:

### Modification of current and future trial designs

At least two large randomized phase III trials in the U.S. need to be modified. The CALGB/SWOG 80405 study compares bevacizumab and cetuximab either alone or together in combination with FOLFIRI or FOLFOX chemotherapy in patients with untreated metastatic CRC. This study, initially designed to enroll 2289 patients, has already accrued about 1400 patients. While the modification plan is still being formulated, it is almost certain that KRAS testing will be required and only patients with wild-type KRAS would be allowed for randomization. A second study, the iBET trial, which was developed based on the BRiTE data, tests whether continuing bevacizumab beyond progression on first line therapy would be beneficial. However, as second line therapy, while patients are randomized to study arms with or without bevacizumab, all patients receive cetuximab. This was probably a forward-thinking design when it was conceived, because the EPIC study showed activity of cetuximab in second line therapy for irinotecan-naïve patients. With more convincing KRAS data, it undoubtedly requires modification. One option would be to completely drop the cetuximab component and to only test the question of whether continuation of bevacizumab beyond progression on first line therapy is beneficial. However, if cetuximab is to be continued as a component in this trial, KRAS testing should be required.

In addition, given the clear predictive value of KRAS status, all future trials involving EGFR-targeted monoclonal antibodies should incorporate KRAS testing.

### KRAS data may or may not predict response to small molecule enzyme inhibitors

The EGFR monoclonal antibodies target the extracellular domain of the receptor. Therefore, tumors with KRAS mutations that confer constitutive activation of intracellular downstream pathways may not respond to these monoclonal antibody-targeted therapies. This may not apply to small molecule inhibitors that specifically target the intracellular downstream protein kinases. At present, small molecule inhibitors have not shown significant benefit in CRCs. However, when more potent agents of this type are developed, they will need to be determined whether their efficacy is influenced by the presence of KRAS mutations in the tumor.

### Therapies for KRAS mutant tumors

The emerging KRAS data leaves open the question of what to do for patients with KRAS mutant tumors. As EGFR monoclonal antibody-targeted therapies are being taken away, these patients are left with only two lines of therapy, either FOLFOX or FOLIRI along with bevacizumab. There is a pressing need to develop novel therapies for this group of patients. Future trials with novel biologically rational agents should be developed for these patients.

### The need for other predictive biomarkers

While data clearly indicate that KRAS MT predicts unresponsiveness to EGFR-targeted monoclonal antibody therapies, KRAS WT does not necessarily predict response: the median response rate of wild-type KRAS tumors in the studies reviewed here was only 35%. Obviously, other markers or combinations of markers that better predict response to a chosen treatment are urgently needed. Markers currently under study include EGFR copy number, EGFR ligands, microsatellite instability (MSI), PTEN, PI3K, and others. However more data will be needed to incorporate these and other novel markers into clinical practice.

### The use of genetic signatures to select treatments

In breast cancer, data have shown that genetic profiling may predict which patients benefit from adjuvant therapy after resection of their breast cancers. It is quite possible that certain genetic signatures will predict response (or lack of response) to certain regimens for CRC. The studies in this field are being actively pursued.

## Conclusion

We have made significant progress in the management of CRC, and to some degree, we have been successful in converting metastatic CRC to a chronic disease state. Targeted therapy, which has been the hot topic for the past decade, now plays a key role in the treatment of CRCs. How to use available agents to maximize the benefit and minimize the associated cost and toxicity is a critical question. Undoubtedly, the future of oncology lies in individualized treatment based on each person's genetic composition. To this end, we have taken a huge step forward with KRAS, and within the next decade, it is likely that we will see more and more biomarkers come into clinical practice to direct individualized treatment.

## Competing interests

The authors declare that they have no competing interests.

## Authors' contributions

DZC, VK, YM, and KL assembled and analyzed the data. SK contributed to discussion. All authors contributed to writing the manuscript. All authors read and approved the final manuscript.
